# Identification of MicroRNA–mRNA Networks in Melanoma and Their Association with PD-1 Checkpoint Blockade Outcomes

**DOI:** 10.3390/cancers13215301

**Published:** 2021-10-22

**Authors:** Robert A. Szczepaniak Sloane, Michael G. White, Russell G. Witt, Anik Banerjee, Michael A. Davies, Guangchun Han, Elizabeth Burton, Nadim Ajami, Julie M. Simon, Chantale Bernatchez, Lauren E. Haydu, Hussein A. Tawbi, Jeffrey E. Gershenwald, Emily Keung, Merrick Ross, Jennifer McQuade, Rodabe N. Amaria, Khalida Wani, Alexander J. Lazar, Scott E. Woodman, Linghua Wang, Miles C. Andrews, Jennifer A. Wargo

**Affiliations:** 1 Department of Surgical Oncology, The University of Texas MD Anderson Cancer Center, Houston, TX 77030, USA; szczepaniak_sloane@yahoo.co.uk (R.A.S.S.); MGWhite@mdanderson.org (M.G.W.); rwitt1@mdanderson.org (R.G.W.); Anik.Banerjee@uth.tmc.edu (A.B.); emburton@mdanderson.org (E.B.); jmgardne@mdanderson.org (J.M.S.); LEHaydu@mdanderson.org (L.E.H.); jgershen@mdanderson.org (J.E.G.); ekeung@mdanderson.org (E.K.); mross@mdanderson.org (M.R.); Miles.Andrews@monash.edu (M.C.A.); 2 Department of Melanoma Medical Oncology, The University of Texas MD Anderson Cancer Center, Houston, TX 77030, USA; MDavies@mdanderson.org (M.A.D.); htawbi@mdanderson.org (H.A.T.); jmcquade@mdanderson.org (J.M.); RNAmaria@mdanderson.org (R.N.A.); swoodman@mdanderson.org (S.E.W.); 3 Department of Genomic Medicine, The University of Texas MD Anderson Cancer Center, Houston, TX 77030, USA; ghan1@mdanderson.org (G.H.); najami@mdanderson.org (N.A.); kwani@mdanderson.org (K.W.); alazar@mdanderson.org (A.J.L.); LWang22@mdanderson.org (L.W.); 4 Department of Biologics Development, The University of Texas MD Anderson Cancer Center, Houston, TX 77030, USA; cbernatchez@mdanderson.org; 5 Department of Medicine, Central Clinical School, Monash University, Melbourne, VIC 3004, Australia

**Keywords:** microRNA, melanoma, immune checkpoint blockade

## Abstract

**Simple Summary:**

We conducted a network analysis of microRNA–mRNA associations in melanoma tissue and cell lines to identify the microRNAs central to melanoma biology and their associated gene expression profiles. Further, we evaluated expression of these microRNAs in melanoma patient biopsies and found that increased expression of miR-100-5p and miR-125b-5p were associated with improved outcomes with anti-PD-1 immunotherapy. Further investigation of these microRNAs as biomarkers and potential targets to improve immunotherapy response in melanoma is warranted.

**Abstract:**

Metastatic melanoma is a deadly malignancy with poor outcomes historically. Immuno-oncology (IO) agents, targeting immune checkpoint molecules such as cytotoxic T-lymphocyte associated protein-4 (CTLA-4) and programmed cell death-1 (PD-1), have revolutionized melanoma treatment and outcomes, achieving significant response rates and remarkable long-term survival. Despite these vast improvements, roughly half of melanoma patients do not achieve long-term clinical benefit from IO therapies and there is an urgent need to understand and mitigate mechanisms of resistance. MicroRNAs are key post-transcriptional regulators of gene expression that regulate many aspects of cancer biology, including immune evasion. We used network analysis to define two core microRNA–mRNA networks in melanoma tissues and cell lines corresponding to ‘MITF-low’ and ‘Keratin’ transcriptomic subsets of melanoma. We then evaluated expression of these core microRNAs in pre-PD-1-inhibitor-treated melanoma patients and observed that higher expression of miR-100-5p and miR-125b-5p were associated with significantly improved overall survival. These findings suggest that miR-100-5p and 125b-5p are potential markers of response to PD-1 inhibitors, and further evaluation of these microRNA–mRNA interactions may yield further insight into melanoma resistance to PD-1 inhibitors.

## 1. Introduction

Immune checkpoint inhibitors targeting cytotoxic T-lymphocyte associated protein-4 (CTLA-4) and programmed cell death-1 (PD-1) have radically improved survival outcomes for metastatic melanoma patients. Treatment with PD-1 inhibitors results in long-term survival for ~40% of patients, compared to ~20% with CTLA-4 inhibition and 5% with the prior standard of care, dacarbazine [1,2,3,4]. However, a significant subset of melanoma patients do not receive clinical benefit from immune checkpoint blockade. Understanding the factors that influence the response to immune checkpoint blockade will facilitate the development of clinically useful biomarkers and new therapeutic strategies to improve patient outcomes.

Translational studies by our lab and others have identified several key determinants of the response to PD-1 inhibition. These include the presence of PD-1 positive T cells and the expression of its ligand, programmed cell death-1 ligand-1 (PD-L1), in the tumor microenvironment (TME), as well as a high tumor mutation burden harboring immunogenic neoantigens [5,6,7,8]. In addition, numerous cellular and genomic parameters have been associated with responses, including distinct transcriptomic profiles, PTEN status, composition of the gut microbiome and the composition of the TME, including levels of B-cells and fibroblasts [8,9,10,11,12,13,14,15,16]. Despite these advances in our understanding, improvements in clinical practice are yet to be realized.

MicroRNAs are post-transcriptional regulators of gene expression, which directly bind to and repress translation of approximately 60% of human mRNAs [17]. MicroRNA regulation of gene expression has an established role in many of the hallmarks of cancer biology [18]. The characterization of melanoma microRNAs has identified disease-specific expression profiles in tissue and serum, including microRNAs with prognostic and predictive value [19,20,21,22]. In addition, specific roles for miRNAs have been identified in diverse biological processes, including angiogenesis, epithelial to mesenchymal transition, invasion, and resistance to targeted therapy [23,24,25,26]. A comprehensive study of The Cancer Genome Atlas (TCGA) Skin and Cutaneous Melanoma (SKCM) dataset (hereafter referred to as “TCGA melanoma”) defined three transcriptomic subsets of melanoma (‘keratin’, ‘MITF-low’ and ‘immune’), each with a distinct microRNA expression profile [27]. Pre-clinical studies of multiple cancer types, including melanoma, have provided evidence of microRNA-mediated immune regulation that can affect sensitivity to immune surveillance [28,29,30,31,32,33,34,35]. However, melanoma microRNA expression has not been extensively studied in the context of clinical immunotherapy responses. We therefore sought to map the landscape of microRNA expression in melanoma and assess associations with immunotherapy outcomes.

In this study, we used a network analysis approach to identify a core set of microRNAs, in TCGA melanoma tumors and patient derived melanoma cell lines, which had strong associations with melanoma gene expression [36]. Using this approach, we identified two distinct microRNA networks, broadly similar to previously identified patterns of microRNA expression in melanoma. We examined the relationship of these microRNAs with survival outcomes in pre-PD-1 inhibitor treatment melanoma biopsies and showed that miR-100-5p and miR-125b-5p, from the same microRNA network, were positively associated with survival benefit.

## 2. Materials and Methods

TCGA melanoma dataset: Normalized mRNA (FPKM) and microRNA (RPM) counts from 368 metastatic melanoma tumors were downloaded from http://gdac.broadinstitute.org/ (accessed on 10 September 2018). We applied a purity filter, which removed samples with <80% tumor nuclei leaving 322 samples for further analysis.

Melanoma cell line dataset: High-purity melanoma cell lines were derived from tumor harvests of melanoma patients participating in The University of Texas MD Anderson Cancer Center’s Adoptive T cell Therapy Clinical Program as previously described [37,38]. Participants provided written informed consent to the collection and use of their tissues for research under protocols (LAB06-0755 and 2004-0069) approved by the Institutional Review Board and in accordance with the Declaration of Helsinki. Normalized mRNA (FPKM) counts were generated from these established melanoma cell lines using the TruSeq Stranded Total RNA LT Sample Prep Kit with Ribo-Zero Gold (Illumina, Inc., San Diego, CA, USA) as per the manufacturer’s instructions followed by 75bp paired-end sequencing on an Illumina HiSeq3000. Reads were aligned to the GRCh37/hg19 genome assembly using STAR (v2.3.0) and gene expression quantified using htseq-count. MicroRNA analysis was performed using the TruSeq Small RNA Library Prep Kit (Illumina, Inc., San Diego, CA, USA) according to the manufacturer’s protocol before sequencing with 35bp single-end reads on an Illumina HiSeq 2500 system. Reads were aligned to miRbase release 20 and mature strand counts derived from longest-match alignments with no mismatches. 

Correlation analysis: MicroRNA–RNA interaction networks: We based our microRNA-mRNA networks on the Spearman correlation of normalized microRNA (RPM) and mRNA (FPKM) count data. We defined a microRNA–mRNA association in the TCGA melanoma dataset as having a Spearman rho <−0.4 using the R function ‘rcorr()’ in the ‘Hmisc’ package (https://cran.r-project.org/web/packages/Hmisc/index.html (accessed on version 4.1-1). In the cell line cohort, the microRNA–mRNA associations were generally stronger allowing us to use a higher Spearman rho threshold (<−0.6) to identify a similar number of microRNA–mRNA associations as the TCGA melanoma dataset. Network visualization and network statistics were calculated using the R package ‘igraph’ (v1.2.4) (https://cran.r-project.org/web/packages/igraph/index.html (accessed on 23 August 2018) [39]. For visualization, only microRNAs with >15 mRNA associations were plotted, full microRNA-mRNA associations are retained in Tables S1–S8.

Bipartite network analysis: Input data for the bipartite network analysis were the Spearman correlation coefficients from the global correlation analysis of microRNA and mRNA expression, as described above, in the TCGA melanoma samples and separately, in the melanoma cell line cohort. MicroRNA–mRNA correlations were filtered to exclude all microRNA–mRNA pairs that did not show inverse correlations (Spearman’s rho <−0.4, <−0.6 in TCGA and cell lines, respectively). Igraph network objects were created from data frames that contained filtered correlation data. Igraph objects were assigned bipartite mapping. The network statistic ‘degree centrality’ was then called for each microRNA in the igraph object, which was then used to filter the microRNAs with the fewest mRNA associations (<20, <100 in TCGA melanoma and cell line datasets, respectively). Incidence matrices of all remaining microRNA–mRNA correlations were generated, and bipartite networks were then projected from the igraph objects.

Unipartite network analysis: Unipartite (one-mode network) igraph objects were generated from the bipartite network analyses described above, using the function ‘bipartite.projection’ from the ‘igraph’ package. Adjacency matrices for each dataset were generated, and the following network statistics were calculated for each microRNA; degree centrality, betweenness centrality, closeness centrality and eigenvector centrality. Unipartite networks were generated using the igraph layout ‘graphopt’.

Gene set enrichment analysis: GSEA was performed through the Broad Institute’s Molecular Signature Database website (https://www.gsea-msigdb.org/gsea/msigdb/annotate.jsp (accessed on 2 September 2018)). Gene set overlaps were compared with the ‘H: Hallmark gene sets’. The top 10 gene sets with an FDR *q*-value < 0.05 are reported.

Melanoma PD-1 cohort: Twenty-two patients with AJCCv8 stage III or IV melanoma undergoing PD-1 immune checkpoint blockade at the University of Texas MD Anderson Cancer Center were included in this study (Table 1). All patient specimens and associated clinical data were collected and used under protocols approved by the Institutional Review Board, and with written informed consent. The selection criteria used for patient identification was having melanoma stage III or IV and having had an adequate biopsy prior to the initiation of immune checkpoint blockade. All patients had cutaneous-type or unknown primary melanoma. Sixteen (73%) patients were male, six (27%) patients were female. Thirteen (59%) patients had prior ipilimumab treatment. Pre-treatment biopsies were collected prior to the commencement of pembrolizumab or nivolumab therapy (median 9.5 days prior to first dose, range 16–231 days) with either no intervening therapy or with a documented continuous progression during any intervening therapy. Best Overall Response (BOR) was calculated using RECIST 1.1 criteria. Nine (41%) patients were classified as having received clinical benefit (BOR; stable disease >6 months, complete or partial response), while thirteen (59%) patients were classified as non-responders (BOR; progressive disease). The measured median progression-free survival (PFS) was 78 days (range; 20–87) in the non-responder and 538 days (range; 321–NA) in the responder groups, respectively.

MicroRNA expression analysis in clinical samples: Total RNA was extracted from snap-frozen, macro-dissected melanoma tumors using the AllPrep DNA/RNA/miRNA Universal Kit (Qiagen), and was quality assessed using the Agilent 2100 Bioanalyzer. For microRNA sequencing, total RNA samples were used as the input for small RNA-sequencing library preparation, using the unique molecular identifier enabled QIAseq miRNA Library Kit (Qiagen). Samples were sequenced using 76 bp single end reads on an Illumina NextSeq 500 and raw UMI count data generated using the QIAseq miRNA analysis pipeline available at geneglobe.qiagen.com (accessed on 6 September 2018). Secondary analysis was performed in R using the DESeq2 package, for a differential expression analysis and count normalisation using the variance stabilizing transformation (vst) method.

MicroRNA survival analysis: Samples were stratified into two groups, either above or below the median expression of the predictor variable. The survival variable of each group, being either time to a progression event (e.g., progression or death), (progression-free survival analysis) or a death event (Overall survival analysis), was then tested using a Cox’s proportional hazard model. Hazard ratios are reported ±95% confidence intervals. Kaplan-Meier curves and log-rank *p*-values comparing overall survival or progression free survival for each variable were generated.

## 3. Results

### 3.1. Landscape of MicroRNA–mRNA Associations in TCGA Melanomas

**Figure 1:**
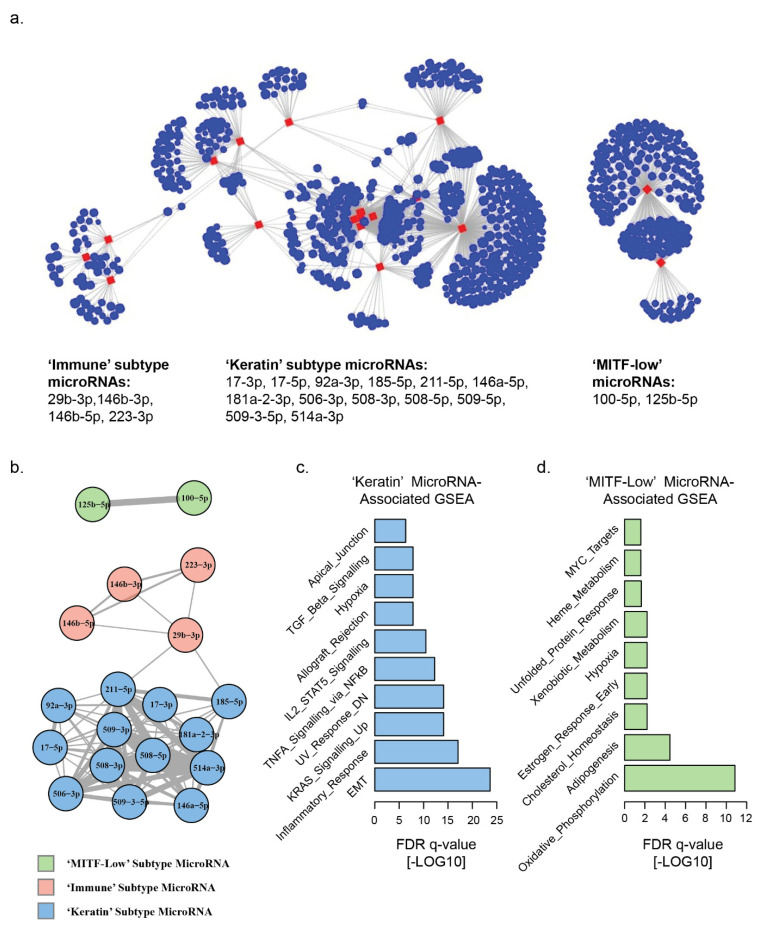
Network analysis of global microRNA–mRNA associations in TCGA melanoma. Inverse correlations of microRNA and mRNA pairs were calculated to identify potential microRNA-regulated gene networks. (**a**) Bipartite network projection based on the 19 microRNAs (red) with the highest numbers (>20) of inversely correlated (Spearman’s rho <−0.4) mRNAs (blue) within all TCGA melanoma samples, identifies three distinct microRNA–mRNA network hubs. (**b**) Unipartite network projection displaying the mRNA inverse correlations shared by each microRNA (a higher number of correlations is indicated by connecting line thickness). MicroRNAs are color coded by their previous association with specific TCGA transcriptomic subsets. (**c**) Gene-set enrichment analysis of all mRNAs inversely correlated with ‘keratin’ transcriptomic-subset-associated microRNAs. (**d**) Gene-set enrichment analysis of all mRNAs inversely correlated with ‘MITF-low’ transcriptomic-subset-associated microRNAs.

We used a network analysis approach to quantify the inverse correlations of microRNA and mRNA expression in the TCGA melanoma dataset [36,39]. We identified 1739 microRNA–mRNA associations, comprising 74 microRNAs which were inversely correlated with the expression of at least one mRNA (Spearman’s rho <−0.4) (Table S1). Of these 74 microRNAs, 19 core microRNAs were associated with >20 mRNAs each, accounting for 1521/1739 (87%) of the total microRNA–mRNA inverse correlations (Table S1, Figure S1a). To further quantify the microRNA–mRNA relationships in our network, we calculated: degree centrality, the number of mRNAs inversely correlated with each microRNA; betweenness centrality which measures how often that node is the shortest path between two other nodes in a network graph; eigenvector centrality, a score derived from the eigenvector of the adjacency matrix of microRNAs, giving higher values to groups of microRNAs with many common mRNA targets. Bipartite and unipartite network projections of the 19 core microRNAs with the highest degree centrality identified three distinct network hubs, which broadly corresponded to the differentially expressed microRNAs in the ‘keratin’, ‘MITF-low’ and ‘immune’ transcriptomic-subsets previously described in the TCGA melanoma dataset (Figure 1a,b) [27]. These three network hubs displayed different levels of interconnectedness, which indicated separate regulatory networks which we further investigated. The largest network hub consisted of microRNAs which were previously found to be differentially upregulated in the ‘keratin’ transcriptomic subset of the TCGA melanoma samples; miR-211-5p, 146a-5p, 181a-2-3p, 506-3p, 508-3p, 508-5p, 509-5p, 509-3-5p, 514a-3p, 17-3p, 17-5p, 92a-3p and 185-5p (Figure 1a,b, Table S2). This network hub accounted for 1153/1739 (66%) of all observed microRNA–mRNA inverse correlations, and contained the microRNAs with the highest degree centrality (miR-211-5p; 293), betweenness centrality (miR-29b-3p, 17-3p, 211-5p, and 185-5p; 42.5, 25.5, 24.5, and 23.5, respectively), and eigenvector centrality (miR-508-3p, 514a-3p, 508-5p, and 509-3p; 1, 1, 0.93, and 0.92, respectively) (Table S1). The second-largest network hub, by number of microRNA–mRNA associations (266/1739, 15%), comprised two microRNAs which were previously found to be differentially enriched in the ‘MITF-low’ cluster of the TCGA melanoma samples: miR-100-5p and 125b-5p. This hub was separate from the rest of the network, having no shared mRNA associations with other microRNAs, and therefore scoring low (<0.01) on eigenvector, and betweenness (Table S1). The third largest network hub, by number of microRNA–mRNA associations (102/1739, 6%), consisted of microRNAs miR-29b-3p, 146b-3p, 146b-5p and 223-3p, which were all previously identified as upregulated in the ‘immune’ cluster of TCGA melanoma samples. This network hub scored low for measures of network centrality (<0.1), indicating very few shared mRNA associations with other microRNAs in the network.

To understand the biological significance of these networks we performed gene-set enrichment analysis (GSEA) of hallmark gene sets from the Molecular Signatures Database on the mRNAs that were inversely correlated with each network (Figure 1c,d, Table S3). The gene set enrichment with the lowest FDR q value in the ‘keratin’ microRNA cluster was the epithelial to mesenchymal transition (EMT) gene set (33 genes, FDR q = 2.36^−24^), consistent with prior experimental evidence of miR-211-5p inhibition of EMT in melanoma [24,40]. The most significant gene set enrichment in the ‘MITF-low’ microRNA cluster was oxidative phosphorylation (15 genes, FDR q = 1.39^−11^). In parallel with the findings of individual mRNA associations, there was also no overlap of gene set enrichment between the ‘keratin’ and ‘MITF-low’ microRNA-associated genes, indicating that these microRNA networks target gene sets with distinct functional potential. No gene sets were significantly enriched in the inversely correlated genes of the ‘immune’ microRNAs, although there were few genes in this group.

### 3.2. Landscape of MicroRNA–mRNA Associations in Patient Derived Melanoma Cell Lines

**Figure 2:**
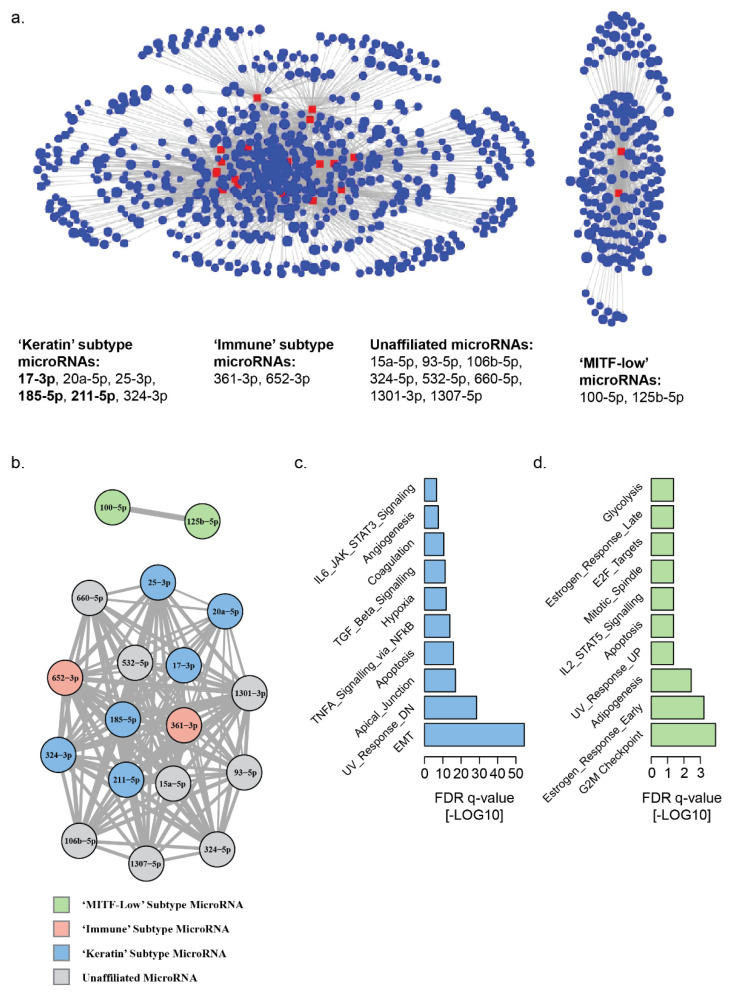
Network analysis of global microRNA–mRNA associations in melanoma cell lines. Inverse correlations of microRNA and mRNA pairs were calculated to identify potential microRNA regulated gene networks. (**a**) Bipartite network projection displaying the 18 microRNAs (red) with the highest numbers (>100) of inversely correlated (Spearman’s rho <−0.6) mRNAs (blue) within all TCGA melanoma samples, identifies two distinct microRNA–mRNA network hubs. (**b**) Unipartite network projection displaying the mRNA inverse correlations shared by each microRNA (a higher number of correlations is indicated by connecting line thickness). MicroRNAs are color coded by their previous association with specific TCGA transcriptomic subsets. (**c**) Gene-set enrichment analysis of all mRNAs inversely correlated with ‘keratin’ transcriptomic-subset-associated microRNAs. (**d**) Gene-set enrichment analysis of all mRNAs inversely correlated with ‘MITF-low’ transcriptomic-subset-associated microRNAs.

To verify our findings from the TCGA melanoma dataset and to clarify the microRNA–mRNA signatures most likely to originate from melanoma cells, we repeated our analysis on a panel of 61 patient-derived early-passage melanoma cell lines from a cohort of patients treated at our institution [37,38]. In this dataset, we identified 4489 microRNA–mRNA associations, involving 81 microRNAs which were inversely correlated with the expression of at least one mRNA (Spearman’s rho <−0.6) (Figure S2a, Table S4). These microRNAs included 2/2 ‘MITF-low’-, 10/13 ‘keratin’- and 1/4 ‘immune’-associated microRNAs that were identified in the original analysis of TCGA melanoma metastatic lesions [27]. Of the 81 microRNAs, 18 were associated with >100 mRNAs each, which accounted for 2748/4489 (61%) of the total microRNA–mRNA inverse correlations (Table S4). Bipartite and unipartite network projections of these 18 microRNAs identified two distinct network hubs (Figure 2a,b and Figure S2b), based on ‘keratin’ and ‘MITF low’ microRNAs. The lack of an ‘immune’-cluster-associated microRNA hub in this cell-line-derived data potentially suggests a reduced contribution from melanoma cells—compared with other cellular elements present in tumors—to the ‘immune’ transcriptomic phenotype. The ‘MITF-low’ network hub comprised the same two microRNAs (miR-100-5p and miR-125b-5p) as were identified in our network analysis of TCGA melanoma tumors and accounted for 308/4489 (7%) of the total microRNA–mRNA inverse correlations in this dataset. This hub was, again, completely separate from the rest of the network (Table S4). The larger hub accounted for 2440/4489 (50%) of the total microRNA–mRNA inverse correlations in this dataset and shared some ‘keratin’ hub microRNAs, including miR-17-3p, 185-5p and 211-5p. However, this hub did not exclusively contain ‘keratin’ microRNAs (6/16), but also ‘immune’ microRNAs (2/16) and eight microRNAs unaffiliated to any TCGA melanoma transcriptomic subtype. The GSEA of the mRNAs which were inversely correlated with the larger network identified striking similarities with the enrichments seen in our analysis of the TCGA melanoma ‘keratin’ network, sharing 6 of the top 10 enriched gene sets, including EMT, UV-response DN, apical junction, TNFA signaling via NFkB, hypoxia and TGF-beta signaling (Table S6). The equivalent GSEA of mRNAs inversely correlated with the ‘MITF-low’ microRNA network in cell lines shared 2/10 enriched gene sets with the TCGA melanoma ‘MITF-low’ network, including estrogen early response and adipogenesis.

### 3.3. PD-1 Treated Patient Cohort

**Figure 3:**
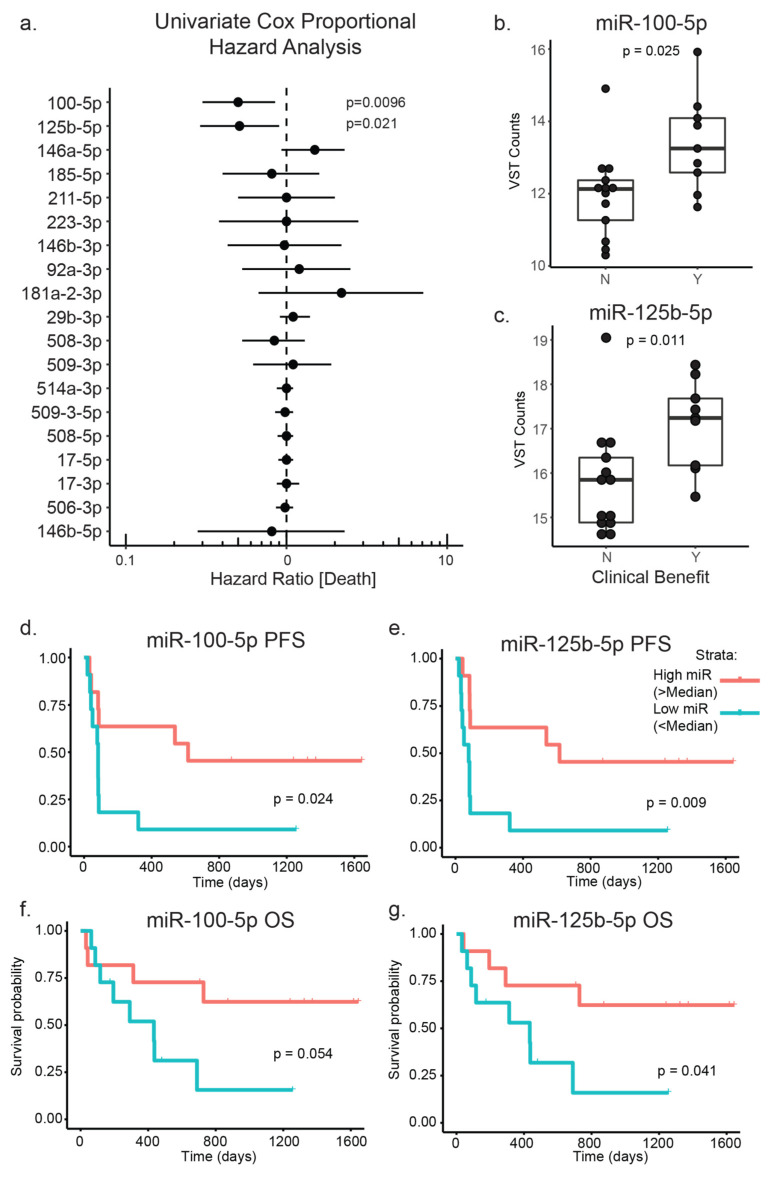
Survival analysis of melanoma microRNAs in pre-PD-1-inhibitor treated melanoma biopsies. MicroRNA sequencing was performed on 22 pre-PD-1-inhibitor-treated melanoma biopsies, and log2-transformed vst counts were generated using DESEq2. (**a**) Forest plot displaying hazard ratios ± 95% confidence intervals, from a univariate Cox’s proportional hazard analysis of each of the 19 microRNAs with the highest degree centrality in the bipartite network analysis of TCGA microRNA–mRNA expression. (**b**,**c**) Boxplots comparing variance-stabilised-log2-transformed counts of miR-100-5p and miR-125b-5p in melanoma biopsies from patients who did not receive clinical benefit from anti-PD-1 immunotherapy versus those who did receive clinical benefit. Boxplots display median, interquartile range and whiskers representing 1.5 times the interquartile range. (**d**–**g**) Kaplan Meier curves displaying the time to PFS or OS for patients with biopsies, with high (above median) compared to low (below median) expression of miR-100-5p or miR-125b-5p. *p*-values represent log-rank p.

Having identified prominent microRNA–mRNA networks in melanoma tumors and cell lines, we sought to validate if certain differentially expressed microRNAs from these networks, measured in melanoma biopsies prior to the PD-1 inhibitor therapy, were related to immunotherapy outcomes. Given the earlier observation that the ‘immune’ transcriptomic subclass was associated with longer post-accession survival of patients with a regional metastatic melanoma in the TCGA melanoma cohort, we hypothesized that microRNAs associated with specific gene expression profiles would be associated with differential responses to immunotherapy. We compared expression of the 19 most highly connected microRNAs identified in our TCGA melanoma bipartite network analysis, in the pre-PD-1 inhibitor treatment biopsies of 22 stage III/IV melanoma patients. Of these 22 patients, nine received clinical benefit (BOR; stable disease >6 months, complete or partial response) and 13 did not (BOR; progressive disease) (Table 1). We then tested all microRNAs for their differential expression, between the clinical benefit and the no clinical benefit groups and observed significantly higher expression of both miR-100-5p (median log2 counts: 12.48 vs. 11.25, *p*-value = 0.036) and miR-125b-5p (median log2 counts: 17.35 vs. 15.49, *p*-value = 0.025) in the tumors of patients who responded to immunotherapy when compared to those who did not (Figure 3a,b, Table S7). Although no other microRNAs were significantly differentially expressed, we did note that miR-146a-5p, which has been implicated as a negative regulator of immune activation in vivo, was slightly elevated in the tumors of patients who did not respond to immunotherapy (median log2 counts: 19.05 vs. 18.13, *p*-value = 0.28) [30]. We then performed a survival analysis using a Cox proportional hazards model and a Kaplan-Meier analysis (Figure 3c–g). The survival analysis showed a lower risk of death which was associated with above-median expression of miR-100-5p (HR [95%CI]: 0.5 [0.3–0.85], *p* = 0.01) and miR-125b-5p (HR [95%CI]: 0.51 [0.29–0.9], *p* = 0.02) (Figure 3c, Table S8), and was most statistically significant for progression-free survival (Figure 3d,e) but also overall survival (Figure 3f,g).

## 4. Discussion

Using a network analysis approach to characterize global microRNA–mRNA relationships in melanoma, we identified three core microRNA–mRNA networks in melanoma tumors that broadly corresponded with the ‘keratin’, ‘MITF-low’ and ‘immune’ transcriptomic subsets which were previously described in the TCGA melanoma dataset [27]. Further investigation of these networks confirmed previous findings about the roles of these microRNAs, including the prominence of miR-211-5p within the ‘keratin’ transcriptomic subset of melanoma and a strong enrichment of epithelial-to-mesenchymal genes, which were inversely correlated with miR-211-5p expression. This supports previous evidence for the regulatory role of miR-211 in EMT-like processes in melanoma [24]. Similarly, we found that miR-100-5p and miR-125b-5p formed an independent network hub, mirroring the association of these microRNAs with ‘MITF-low’ melanomas from previous TCGA analyses [27]. The mRNA targets of the ‘MITF-low’ hub, including miR-125b, showed enrichment for involvement in oxidative phosphorylation, which is consistent with prior evidence implicating miR-125b as a regulator of mitochondrial metabolism [41,42,43], and numerous studies suggesting lower proliferative activity of melanoma cells in ‘MITF-low’ phenotypic states [44,45,46].

The network analysis of the melanoma cell lines shared broad similarities with the findings in TCGA melanoma tumor samples, with a high degree of overlap in terms of individual microRNA–mRNA associations, and in the gene set enrichments of those mRNAs. The strongest agreement between tumors (TCGA) and cell lines was observed for the miR-125b and miR-100-5p network, being evident in both datasets as a distinct miRNA hub without overlap of other microRNA–mRNA pairs. However, one notable difference in this network, in the cell lines, was the absence of the enrichment of the oxidative phosphorylation gene set in genes inversely correlated with miR-100-5p and miR-125b-5p, possibly reflecting differences in the metabolic requirements between cell culture and the intact TME, or that these metabolic differences are attributable to non-melanoma cellular components of the TME such as monocytes [41,47,48]. These findings nonetheless implicate cellular energetics as a key potential therapeutic target in ‘MITF-low’ melanomas, as modulators of a metabolically permissive microenvironment.

The correlation analysis within the melanoma cell line dataset confirmed key findings from our network analysis of the TCGA melanoma dataset, however, several differences were noted, including a substantially higher number of microRNA–mRNA pairs. This discrepancy may have been for several reasons, including due to the sampling of high purity cell lines with consistent culture conditions, compared to the variability inherent in whole tumors which have unpredictable stromal, immune and metabolic variations, and an established difference in microRNA expression in cell culture [48]. Due to these differences we chose to use the microRNAs identified in the TCGA melanoma dataset for additional survival analyses in our tumor specimens.

Using a Cox’s univariate proportional hazard model, we found that both ‘MITF-low’ microRNAs from our network analysis, miR-100-5p and miR-125b-5p, were associated with a clinical benefit in our PD-1 treated cohort. Interestingly, previous research has implicated the expression of several microRNAs, including these, in myeloid-derived suppressor cell (MDSC)-mediated resistance to immune checkpoint inhibitors. It is important to note that in that study, microRNAs were measured in peripheral blood plasma samples, and it is therefore unclear how these compare with the levels measured in tumors [49]. Although there is limited existing experimental evidence for the role of these microRNAs in melanoma immunity, it should be noted that the gene network surrounding these microRNAs revealed a strong depletion at the gene set level of oxidative phosphorylation, a pathway which has recently been implicated in melanoma immune evasion in melanoma brain metastases [50]. Ultimately, these preliminary findings will require future validation in a larger cohort, as our limited sample size is a major limitation of this study. Beyond validation of the two microRNAs of interest, limitations of sample size can potentially lead to a type two error. Further exploratory analyses of larger cohorts studied may lead to additional microRNAs of interest. Additionally, further work is needed to define more clearly the role of MITF-low-expressing states in primary or adaptive resistance to immune checkpoint blockade in melanoma.

Beyond the ‘MITF-low’ microRNAs, we found a trend towards higher miR-146a-5p expression in melanomas that did not receive clinical benefit, although this result did not reach statistical significance, possibly due to the small cohort size. This aligns with a pre-clinical model of miR-146a-5p in melanoma associated with a resistance to immunotherapy, and also highlights a potential dichotomy between ‘keratin’ and ‘MITF-low’ associated microRNAs and immunotherapy responses [30].

## 5. Conclusions

We characterized microRNA–mRNA networks in melanoma tissue and cell lines consistent with the current understanding of ‘keratin’ and ‘MITF-low’ melanoma transcriptomic subtypes. This analysis identified known and novel microRNA–mRNA associations in melanoma tissues and cell lines, including EMT, signal transduction and metabolic pathways associated with distinct microRNA networks. Furthermore, in a cohort of pre-PD-1 treated melanoma biopsies we found an association between the ‘MITF-low’ microRNAs, miR-100-5p and miR-125b-5p, with clinical benefit from PD-1 checkpoint blockade. Despite this and other advances in our understanding of checkpoint blockade, no single factor has been delineated that encompasses the complexity of these malignancies. As has been noted, as these single biomarkers are described, they may ultimately have the most clinical utility within a composite score, to predict the responses to immuno- or other therapies [51].

## Figures and Tables

**Table 1:** Clinical characteristics of the PD-1 inhibitor treated patient cohort.

Characteristic	PD-1 Inhibitor No Clinical Benefit (*n* = 13)	PD-1 Inhibitor Clinical Benefit (n = 9)
Sex		
Male	8 (62%)	8 (89%)
Female	5 (38%)	1 (11%)
Melanoma Type		
Cutaneous unspecified	5 (38%)	4 (44%)
Superficial spreading	2 (15%)	-
Nodular	4 (31%)	-
Acral lentiginous	1 (8%)	1 (11%)
Unknown primary	1 (8%)	4 (44%)
Disease State (AJCCv8)		
IIIa/b	-	-
IIIc/d	2 (15%)	3 (33%)
IVa	2 (15%)	-
IVb	1 (8%)	-
IVc	8 (62%)	6 (67%)
IVd	-	-
Elevated Serum LDH (*n* (%) > ULN)	6 (46%)	5 (56%)
Prior Ipilimumab		
Yes	9 (69%)	4 (44%)
No	4 (31%)	5 (56%)
Best Overall Response (BOR, RECIST 1.1)		
CR	-	3 (33%)
PR	-	4 (44%)
SD	-	2 (22%)
PD	13 (100%)	-
PFS (median, range; days)	78 (20–87)	538 (321–NA)

## Data Availability

Genomic data are available from the European Genome-Phenome Archive (EGA) under accession EGAS00001004536 upon valid request to the applicable Data Access Committee as indicated via the EGA. The data presented in this study are available on request from the corresponding author. The data are not publicly available due to patient privacy restrictions.

## Supplementary Materials

The following are available online at https://www.mdpi.com/article/10.3390/cancers13215301/s1, Figure S1: Network analysis of global microRNA–mRNA associations in TCGA melanoma samples, Figure S2: Network analysis of global microRNA–mRNA associations in melanoma cell lines, Table S1: TCGA microRNA: mRNA bipartite and unipartite network statistics, Table S2: Incidence matrix of all statistically significant microRNA: mRNA correlations in TCGA dataset, Table S3: Gene-set enrichment analysis of hallmark gene sets from the Molecular Signatures Database in TCGA microRNA: mRNA networks, Table S4: Melanoma cell line microRNA: mRNA bipartite and unipartite network statistics, Table S5: Incidence matrix of all statistically significant microRNA: mRNA correlations in melanoma cell line dataset, Table S6: Gene-set enrichment analysis of hallmark gene sets from the Molecular Signatures Database in melanoma cell line microRNA: mRNA networks, Table S7: MicroRNA differential expression analysis between the clinical benefit and the no clinical benefit groups, Table S8: Survival analysis of microRNAs in PD-1 inhibitor treated melanoma patients.
