# Supplementary figures and images for: Identification of MicroRNA–mRNA Networks in Melanoma and Their Association with PD-1 Checkpoint Blockade Outcomes
Source: Cancers (Basel). 2021 Oct 22;13(21):5301. doi: 10.3390/cancers13215301 (PMC8582574; doi:10.3390/cancers13215301)

a.

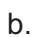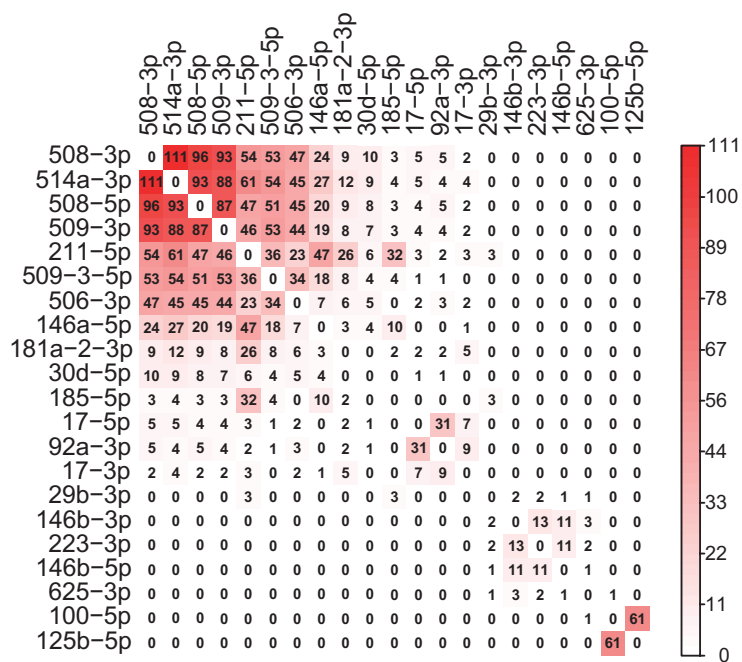

Figure S2. Network Analysis of Global MicroRNA:mRNA Associations in Melanoma Cell Lines.

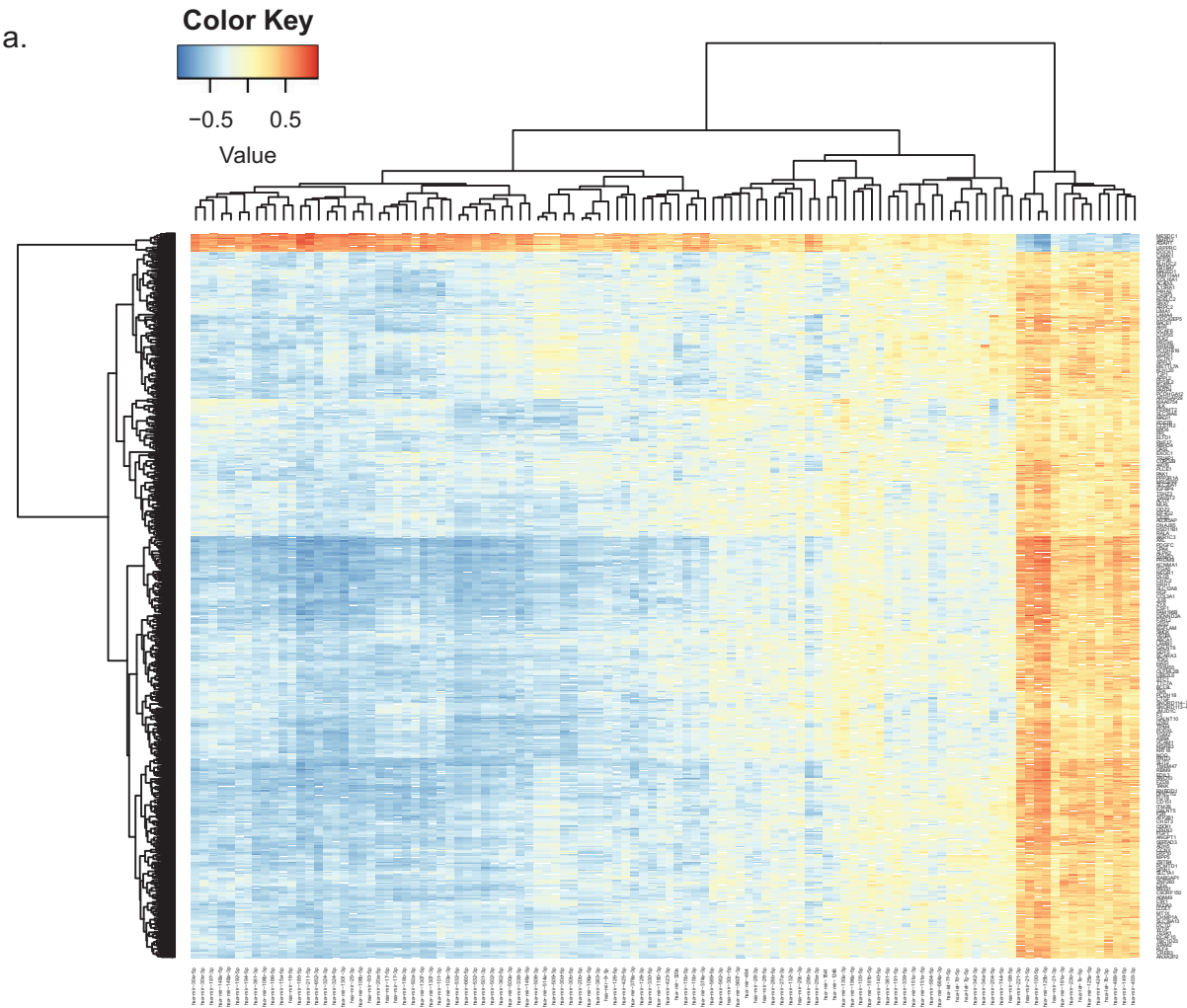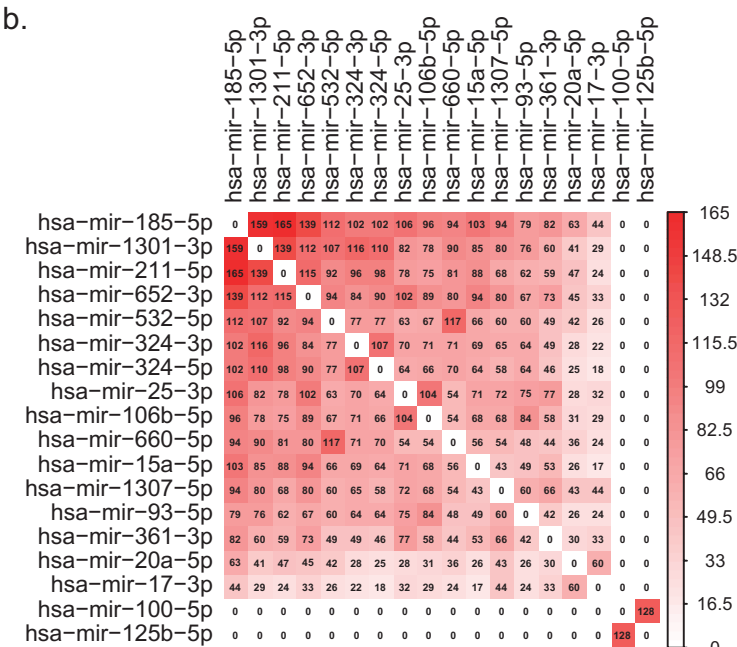

Supplement: Supplementary file 1 [file cancers-13-05301-s001.zip › Supplemental Figures.pdf]
